# Social organization of necrophoresis: insights into disease risk management in ant societies

**DOI:** 10.1098/rsos.240764

**Published:** 2024-12-11

**Authors:** Quentin Avanzi, Léon Lisart, Claire Detrain

**Affiliations:** ^1^ Unit of Social Ecology, Université Libre de Bruxelles, Brussels, Belgium

**Keywords:** social immunity, *Myrmica rubra*, *Beauveria bassiana*, generalist entomopathogen, work organization, waste management

## Abstract

Insect societies, which are at a high risk of disease outbreaks, have evolved sanitary strategies that contribute to their social immunity. Here, we investigated in the red ant *Myrmica rubra,* how the discarding of nestmate cadavers is socially organized depending on the associated pathogenicity. We examined whether necrophoresis is carried out by a specific functional group of workers or by any nestmates that may become short-term specialists. By observing the behavioural profiles of tagged individuals, we assigned half of the colony members to functional groups (foragers, intermittent-foragers, domestics, nurses and inactives). Following the introduction of uninfected or sporulating corpses into the nest, intermittent-foragers were the functional group most involved in necrophoresis, as they touched, moved and discarded more cadavers. Interestingly, sporulating corpses induced a more generalized response in workers from all functional groups, thereby accelerating their rejection from the nest. The individuals contacting corpses were also prophylactically engaged in more grooming behaviour, suggesting the existence of hygienist workers within ant colonies. These findings raise questions about a trade-off existing between concentrating health risks on a few workers who are highly specialized in necrophoresis and exposing a larger population of nestmates who cooperate to speed up nest sanitization.

## 1. Introduction

Work organization emerges from variations in response thresholds to task-related stimuli among workers who have access to local information and follow simple behavioural rules [1,2]. Behavioural thresholds vary among nestmates depending on their physical caste in polymorphic species [3], age [4,5], physiology [6], genetic background [7] and/or individual experience (pre-imaginal: [8,9], adult: [10]). Furthermore, task specialization usually occurs among individuals showing not only the appropriate response threshold but also the appropriate location close to the tasks to be fulfilled [11]. Ergonomic plasticity, that is the capacity of a colony to cope with workload fluctuations, can be achieved by adjusting the ratio of workers allocated to different types of activities and/or the level of individual investment in a given task [12–14]. Adjustments in task allocation can also operate on different timescales, from a short-term and reversible specialization of certain individuals in sporadic tasks (in the case of necrophoresis [15–17]) to a colony-level change in the proportion of nestmates that specialize in some specific tasks for most of their lifetime [18]. In polymorphic species, changes in work organization can even take place on an evolutionary timescale, with the ratio of each caste being determined by selective pressures operating on the ant species [19].

Task allocation and its regulation determines the ability of a colony to respond to changing conditions as well as of the time and/or energy required to meet colonial internal needs, such as brood care or food sharing. Flexibility and efficiency in performing hygienic tasks are other major assets for which ants have evolved prophylactic and hygienic behaviours to limit health problems caused by pathogens [20]. These adaptive sanitary strategies, which are referred to as ‘social immunity’ [20,21], aim to reduce exposure to pathogens (avoidance strategy), decrease their load within the colony (resistance strategy) or compensate for their negative impacts on colony fitness without reducing the pathogen load (tolerance strategy). Workers may also exhibit spatial fidelity and adapt interaction networks [22] to limit the spread of potential pathogens through organizational immunity [23]. Given that faeces, food remains and cadavers are potential sources of pathogens [24], the management and disposal of these wastes outside the nest are basic components of resistance sanitary strategies and prophylaxis. As with any task essential for colony fitness, waste management should ideally rely on an efficient work organization that maximizes the speed of waste removal and minimizes the costs associated with mortality risk [20]. For example, the handling of pathogenic waste by older individuals reduces the costs resulting from the death of workers with shorter life expectancies [25]. The incurred risks can also be limited to a few individuals that become temporarily specialized in waste management, including the discarding of nestmate corpses (in *Myrmica rubra* [17]). This short-term specialization enables the colony to respond quickly to fluctuations in mortality risks while avoiding the maintenance of long-term specialists that would remain inactive most of the time, except in rare events of massive mortality.

Necrophoresis, also known as undertaking, consists of discarding dead nestmates [26], usually further away from the nest than inert items [27]. Ant societies adapt waste management to their associated pathogenicity [28,29]. For example, workers of the red ant *M. rubra* reject fungus-contaminated items or sporulating insect corpses at higher rates than their uninfected counterparts [30–32]. It is often assumed that faster task completion results from a higher degree of individual specialization, as in the case of foraging [33]. However, the benefits derived from task specialization are more complex to predict in the case of sanitary tasks because of the multiple levels of selective forces at work. At the individual level, if some workers are more frequently exposed to or more prone to displace infected cadavers, these specialists will experience high pathogen exposure and high mortality rates. Nevertheless, it is possible that these ants, which engage in corpse management, possess a higher level of individual immunity compared with other workers [34]. At the colony level, costs associated with waste management remain limited due to the small number of these specialists exposed to sanitary risks. An alternative strategy can occur if a large number of non-specialized workers are involved in waste management. Then, the colony will increase the likelihood of detecting infectious items and speed up nest sanitation, but at the same time, this will increase the proportion of the worker population at risk of morbidity. Furthermore, any worker committed to waste management could become a ‘vector’ for pathogens or even a ‘hub’ for transmission if not socially secluded [23,35]. The number of workers involved in waste management, and hence the level of specialization, is thus expected to vary with waste pathogenicity, as it can profoundly influence the risk of disease outbreaks and the ultimate mortalities at the colony level.

In this study, we investigated the extent to which individuals from different functional groups participated in each step of necrophoresis, from the initial detection of cadavers to their discarding from the nest. We defined a functional group as a group of workers that perform the same tasks over an extended period of time and usually share the same location. In the case of necrophoresis, the probability that a worker will engage in corpse management is expected to depend on both the spatial location of the worker and its response threshold to corpse-related stimuli. Therefore, we hypothesized that functional groups that are predominantly located inside the nest will be the first to come into contact with cadavers and displace them towards the nest entrance, at the edge of their spatial zone. We then anticipated that, similar to previous studies on uninfected cadavers [15,16], corpses will be discarded by workers who are usually involved in external tasks. We also investigated whether the faster discarding of infectious items (as previously shown by [30]) is due to a few specialists being particularly efficient in the disposal of hazardous items, or whether it results from a large number of non-specialized workers involved in waste management. Since a limited number of specialists may reduce the discovery rate of waste and leave valuable individuals such as brood and queen unprotected for a long time, we speculated that, in the case of sanitary emergencies (e.g. the presence of sporulating corpses), the balance will be tipped towards a reduced level of individual specialization in order to speed up the discarding of hazardous items and ultimately reduce the exposure of the entire colony to pathogens.

Finally, we assessed whether the social organization of necrophoresis can be related to differences in the mortalities of individuals, functional groups and colonies as a whole. If infectious corpses are discarded by ant specialists, the number of exposed individuals is limited. On the other hand, the pathogen load *per capita* and hence individual mortality risks are probably higher than if this threat is managed by a large number of unspecialized workers [36]. Besides, since mutual or self-grooming allows the reduction of pathogen load over the body of ant workers [37–39], we investigated whether ant societies contain hygienist workers who perform above-average prophylactic grooming prior to any exposure to pathogens and whether these individuals belong to the group of workers most involved in necrophoresis.

## 2. Material and method

### 2.1. Biological models

The ant species, *M. rubra,* is commonly used as a biological model in studies about social immunity [16,17,40]. *Myrmica rubra* is a common ant species in temperate areas of Europe and is considered an invasive ant in North America. This polygynous species usually lives in semi-open forests and grasslands [41], with nest populations ranging from 1 to 20 queens and 100 to 2500 workers in Europe [42]. Its nests are dug in various substrates, such as soil under stones, rotting wood or roots of nettles and bramble bushes ([42], personal observations). Although ants share confined nest sites with genetically related nestmates, disease outbreaks leading to the death of the whole colony are rarely observed [43]. However, as an omnivorous species that frequently forages on prey, *M. rubra* workers are exposed to insect remains, which can be a source of entomopathogens. The ant genus *Myrmica* is actually exposed to a wide variety of parasites [44] and is commonly confronted *in naturae* to the generalist entomopathogenic fungus *Beauveria bassiana* [44,45]. Infection by this fungal pathogen starts with the attachment of conidia, i.e. asexual propagules, on their cuticle, and by the release of an enzymatic cocktail allowing a germ tube to penetrate the cuticle and enter the insect’s body [46]. The fungal mycelium then grows and invades the haemocoel, leading to the death of the host [47]. Ultimately, the fungus works its way outside the insect body to produce sporulating structures bearing conidia propagules [46]. At the last stage of infection, conidia are produced over the host cadaver, resulting in the white fluffy appearance of *B. bassiana* sporulating corpses, also called ‘white muscardine’ [46].

### 2.2. Collection and rearing of ant colonies

Five *M. rubra* colonies were collected during the summer of 2021 in woods located at Aiseau-Presles (Province of Hainaut: 50°25.657′ N; 04°35.674′ E) and Sambreville (Province of Namur: 50°25.210′ N; 04°37.878′ E). Nests were dug out from the litter and birch wood logs. Ant colonies were maintained in a room with constant temperature and humidity (21 ± 1°C and 50 ± 5%) and 12 h : 12 h dark : light regime and darkened test tubes were offered as nesting sites.

From these colonies, we made five experimental colonies consisting of two queens, 225 workers (around one-third foragers and two-thirds internal workers), and 60 larvae at the second and third instars. Each experimental colony was taken from a different mother colony. Foragers were sampled from among ants walking outside the nests, and internal workers were removed from nesting test tubes. These ants were transferred to an experimental tray (30 × 22 × 7.5 cm), the bottom of which was covered by a 1 cm plaster layer, and the edges were coated with Fluon^TM^ to prevent ants from escaping. A hole was drilled in the tray, vertical to the experimental nest. A cotton wick was inserted into the hole and placed over a sponge soaked daily to evenly moisten the plaster below the nest.

**Figure 1.**
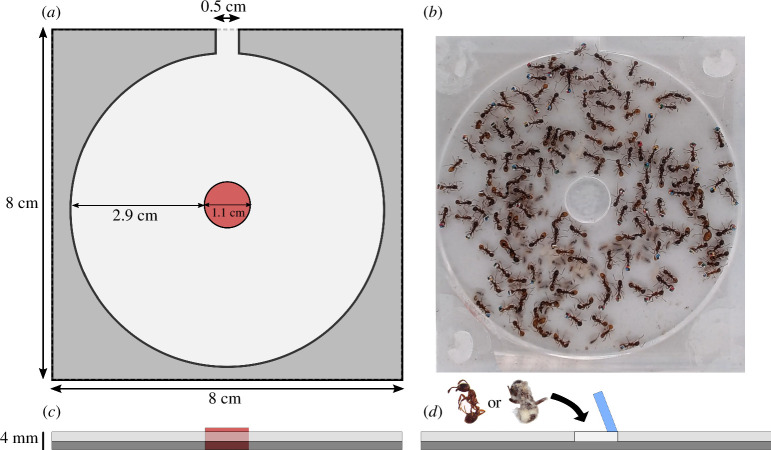
Diagram of the nest from different perspectives. *(a)* Top view. *(b)* Screenshot of a video used during behavioural analyses. *(c,d)* Side view of the nest prior to the introduction of corpses *(c)* and during their introduction *(d)*. In red *(a,c)*, the mobile column inserted in the centre of the nest allows to free a space for the further introduction of corpses. In blue *(d)*, the mobile plexiglass plug that closes the roof opening during the observation of necrophoresis.

The experimental nest was circular (6.9 cm diameter) with a single chamber and a single entrance (5 × 5 × 2 mm). It was made of two square Plexiglas plates (8 cm on each side and 2 mm in thickness). The gap between these two plates created a 2 mm high space between which ants settled in a monolayer, thus facilitating behavioural observations (figure 1). The nests were designed in vector drawings using the InkScape software and cut out using a laser cutter (High-Z S-1000/T CNC Router). The nest roof plate had a central circular hole (1.1 cm diameter) that could be alternately closed by one of the following two removable parts. The first removable part (red in figure 1*a*) was a column passing through the roof down to the plaster ground (1.1 cm radius × 4.1 mm high). This created a space in the centre of the nest where workers could not settle and where corpses could be further dropped inside the nest with minimal disturbance to nestmates (see §2.3). The second removable part (blue in figure 1*d*) was a transparent circular plug that served as a roof cover during the 4 hours of observations following the insertion of corpses. This plug allowed for the passage of ants underneath and provided them with access to the inserted items. Finally, the nest was surrounded by a plexiglass cage coated with Fluon (polytetrafluoroethylene) to prevent the ants from climbing above the nest and disturbing behavioural observations. Experimental colonies were provided with water, a 0.3M sucrose solution and 10 dead fruit flies (*Drosophila melanogaster*) were placed daily in the area at 9:30. Fruit flies were chosen as the protein source because they were small enough to be retrieved by ants through the nest entrance and large enough to observe prey consumption by ants inside the nest.

### 2.3. Experimental procedure

#### 2.3.1. Planning

**Figure 2.**
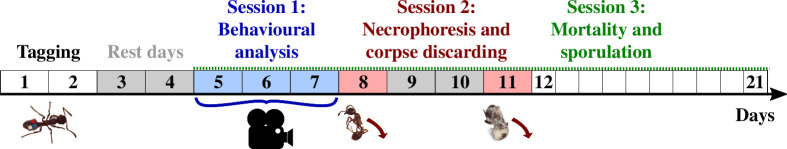
Experimental procedure. The study included three experimental sessions represented in blue, red and green. Days 1 and 2: tagging of workers. Days 3 and 4: rest period to stabilize the ant population inside the nest and to habituate workers to the tags. Days 5 to 7: recording of tagged workers behaviour inside the nest (three times per day for 3 min). Day 8: introduction of 10 uninfected cadavers in the nest and observation of necrophoresis by ants. Days 9 and 10: rest period before the second introduction of cadavers. Day 11: introduction of 10 sporulating cadavers in the nest and observation of necrophoresis. Days 5 to 21: each day, dead workers were counted, taken out of the colony and put in sporulation.

The experiment lasted for three weeks for each tested colony (figure 2). It began with 2 days of tagging, followed by 2 days of settlement and habituation of the ants to the experimental set-up. It was organized into three distinct experimental sessions. During the first session, the behaviour of tagged ant individuals inside the nest was quantified for 3 days to further assign them to a functional group. The second session consisted of introducing uninfected corpses and, 2 days later, sporulating dead nestmates. This allowed us to quantify the dynamics of corpse removal. Particular attention was paid to the interaction of each tagged ant with corpses to assess whether their contribution to necrophoresis differed according to their functional group. During the third session, we monitored daily mortality and the causes of death of workers by placing their cadavers under sporulation conditions. This monitoring started on day 5, at the same time as session 1, and continued until day 21 (figure 2).

#### 2.3.2. Tagging

Around half of the worker population in each experimental colony was tagged individually. The tags were made of waterproof ‘toughprint’ paper (0.9 mm^2^ square) marked with four tiny 0.45 × 0.45 mm coloured squares. One square was black and the other three had a combination of different colours, selected to maximize contrast on video recordings (blue, green, red, white and yellow, figure 1). This allowed us to identify the ant individual, regardless of its position, simply reading the colours clockwise, starting with the black square.

Before being tagged, the ants were briefly CO_2_-anaesthetized for 45 seconds. To restrict their movements, the ant head and thorax were delicately placed in slits made of a professional make-up sponge (Cala^TM^). A droplet of glue (Loctite^TM^ superglue) was applied to the first abdominal segment using a small guitar string (D’Addario^TM^ NYS007), and the coloured tag was placed using a toothpick (see electronic supplementary material). While being removed from the slit, the tagged ants were briefly anaesthetized again for 1 min and then isolated in Petri dishes until the glue dried completely. Four hours later, the tagged ants were placed in the experimental tray along with their conspecifics. As approximately 20% of the tagging attempts failed, we tagged 125 workers to ensure that we had approximately 100 tagged workers per colony at the beginning of the experiment (see electronic supplementary material for more details). The ants were randomly selected for tagging in a ratio of two-thirds internal to one-third external workers. We excluded callows or any individual showing signs of injury. In total, 491 workers were tagged successfully. As shown in previous studies, tagging had no significant effect on a worker’s behaviour [48–50], except for a temporary increase in self-grooming behaviours that lasted no longer than 24 hours.

#### 2.3.3. Session 1: Behavioural profile and assignment to a functional group

For each tagged individual, we quantified its location (outside versus inside the nest) as well as its behaviours inside the nest by observing it three times (10.30, 13.30 and 16.30) for 3 min over 3 successive days (figure 1). The recorded behaviours are listed in table 1. On video recordings, we quantified the proportion of time each tagged ant was engaged in a given behaviour over the total duration during which this ant was observed inside the nest. The time that a tagged worker spent outside the nest was considered as foraging. Inside the nest, if a behaviour stopped for less than 5 s, it was considered uninterrupted. If an ant was motionless and did not engage in any activity for at least 5 s, it was recorded as inactive (based on the same criteria as in [52] study). Any instance of grooming behaviour performed by a worker alone, cleaning any part of its body such as antennae or abdominal tip, was classified as ‘self-grooming’. Similarly, when a worker actively groomed a nestmate, it was reported as ‘allogrooming’. Once the data were collected, we compiled a behavioural profile for each tagged worker. The proportion of time spent on each task was used in a cluster analysis to assign each worker to one of the functional groups (see §2.4 for further details).

**Table 1.** List of behaviours observed during the video analysis (adapted from [51]).

behaviour	definition
foraging	located outside of the nest, in the foraging area
moving inside	mobile inside of the nest without being engaged in another task
trophallaxis	receive or give liquid food to/from another ant
eating	feeding on a drosophila inside the nest
brood care	manipulating brood (feeding, grooming and transporting)
self-grooming	grooming itself or performing extensive grooming
allogrooming	grooming another ant
inactivity	immobile and not engaged in any other task for at least 5 s

#### 2.3.4. Session 2: Necrophoresis

We compared the behaviour of tagged individuals exposed to either 10 uninfected or 10 sporulating corpses. The 10 uninfected cadavers were nestmates killed by freezing 1 day before their corpses were introduced into the nest (figure 1). Indeed, it takes 24 h for the cuticular profile of dead workers to become enriched in oleic and linoleic acids, which allows their recognition as cadavers and triggers necrophoresis [53,54]. The 10 sporulating cadavers were nestmates that died from infection by the entomopathogenic fungus *B. bassiana* (Naturalis-L^TM^). These sporulating corpses were produced prior to the start of the experiment. To do so, we topically applied a droplet of Naturalis-L insecticide (±4 µl) to the abdomen of 30 nestmates that were isolated in a Petri dish with a wet cotton ball until their death. On the day of their death, ant cadavers were washed with ethanol and distilled water according to the Lacey method [55] to avoid contamination by microorganisms other than *B. bassiana* fungus. Following this treatment, the cadavers were placed on moist filter paper in a Petri dish sealed with parafilm and placed in a temperature-controlled cabinet at 25°C until the fungus sporulated over the ant body.

As for the course of the necrophoresis experiment, 10 uninfected cadavers were introduced on day 8 in the centre of the experimental nest (figure 1). After delicately removing the column from the nest (figure 1, in red), in a fast and smooth movement, we dropped the cadavers through the hole and immediately closed them with the circular plug (figure 1, in blue). As video recordings were launched before the introduction of corpses, we could observe the first workers who came into contact with corpses. Workers’ behaviour was filmed continuously for 4 h (10.00 to 14.00) and thereafter for 3 min every half hour for another 4 h. After the last cadaver was discarded outside the nest, the roof plug was gently replaced by the column piece in order to create an empty space for the second insertion of sporulating bodies. After 2 days of rest (figure 2), 10 sporulating cadavers were introduced on day 11 following the same procedure as described above. For both uninfected and sporulating corpses, we measured the time elapsed before they were removed from the nest, the identity of the workers that interacted with them, and the time, number and type of interactions that each tagged worker had with the corpses. Any interaction lasting more than 2 s was recorded as contact, regardless of whether the ant touched the corpse with its antennae or mandibles. Interactions lasting less than 2 s were not considered. If the worker grabbed the corpse with its mandibles and moved it from its location, the interaction was considered as a displacement. The last displacement of a corpse that carried the cadaver outside the nest was considered as a discarding.

#### 2.3.5. Session 3: Mortality and sporulation

From days 5 to 21, the bodies of the dead workers were removed from the tray each morning. For each cadaver, we identified the dead worker by its tag (if present), and we determined the cause of death by placing it in sporulation following the Lacey method, as described above [55]. The sporulation status of the corpses was monitored for two weeks by placing them in a dark, thermoregulated cabinet at 25°C.

### 2.4. Statistical analyses

All statistical analyses were conducted using RStudio software (RStudio Desktop v. 1.3.1056). The graphs and tables were created using Excel and R (*ggplot2* and *flexplot* functions). The R code used to perform these analyses is provided in the electronic supplementary material for the reader’s reference.

#### 2.4.1. Behavioural profile and assignment to a functional group

First, in order to determine the functional groups, behavioural data from all the tagged individuals from the five colonies were pooled to perform a principal component analysis (PCA) (*stats* package, *princomp* function). Based on the results of this analysis and data from the existing literature [6,51,56–60], five different functional groups were identified: foragers, intermittent-foragers, domestics, nurses and inactives (see §3).

Second, in order to assign each tagged individual to one of the five functional groups, we used the dataset from its own colony, as this is the relevant level of study when examining work organization. As brood care was observed to be a relatively rare behaviour, workers that spent at least 5% of their time taking care of brood were defined as nurses. All other tagged workers were assigned to one of the four other functional groups by means of a centroid cluster analysis performed on their colony dataset (*factoextra* package, *kmeans* function). The procedure resulted in a well-balanced proportion of each of the five functional groups in each of the five colonies (for further details, refer to the supplementary material).

To test for possible changes in the behavioural profiles of tagged workers over the 3 days of observation, a Friedman test (*friedmann.test* function) was conducted, followed by post hoc analysis using pairwise Wilcoxon–Mann–Whitney tests (*pairwise.wilcox.test* function). We also conducted Kruskal–Wallis tests to compare behavioural profiles across colonies (*kruskal.test* function), followed by post hoc Nemenyi tests (*kwAllPairsNemenyiTest* function). Further details are provided in the electronic supplementary material, figure S3.

**Figure 3.**
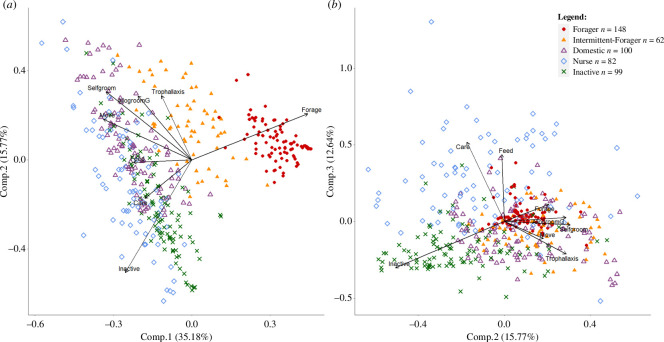
Principal component analysis of all behavioural profiles. (*a*) The first component allows to differentiate between internal workers (left), ‘intermittent-foragers’, and ‘foragers’ (right). (*b*): Components 2 and 3 allow to differentiate between ‘nurses’ (top), ‘inactives’ (bottom left) and ‘domestics’ (bottom right). Note that the assignment of tagged workers to one of the five functional groups was done at the colonial level using a centroid clustering method (see electronic supplementary material).

#### 2.4.2. Necrophoresis

The number of contacts with cadavers was compared across functional groups with a Kruskal-Wallis test followed, if significant, by pairwise comparisons using the Nemenyi test. Owing to the many tied values, we used a correction procedure by randomizing the rank of those ties 50 times and keeping only the highest *p*-value obtained (*rank* function, ties.method = random). The proportion of tagged individuals contacting corpses or not was tested in each functional group using a *χ*^2^-test followed by pairwise *χ*^2^ independence tests with ‘false discovery rate’ correction as post hoc tests (functions: *chisq.test* and *pairwiseNominalIndependence*, method = *fdr*). The same statistical analyses were applied to the proportion of tagged individuals in each functional group that displaced corpses (functions: *chisq.test* and *pairwiseNominalIndependence*, parameters; chisq = *T*, method = *fdr*). Since the same tagged workers were exposed to non-infected and sporulating corpses, the proportion of individuals contacting/displacing cadavers were compared between those two conditions using McNemar test (*mcnemar.test* function).

#### 2.4.3. Mortality and prophylactic grooming

The survival curves were compared across functional groups using Kaplan–Meier tests (*survfit* function). The same test was employed to control for the colonial effect on survival curves, with no significant differences observed between the five colonies (electronic supplementary material, figure S5, Kaplan–Meier, n.s.). To assess whether different levels of exposure to the corpses had an impact on the mortality of workers, we performed *χ*^2^-test followed whenever needed by pairwise *χ*^2^ independence tests as post hoc (functions: *chisq.test* and *pairwiseNominalIndependence*, method = *fdr*).

Wilcoxon tests were also conducted to determine whether workers who contacted corpses exhibited higher levels of self-grooming or mobility, prior to the introduction of cadavers, than workers that did not contact them (*wilcox.test* function). Finally, we tested the potential correlation between the time a worker spent grooming itself and its mobility using the Pearson correlation (*ggscatter* function, cor.method = ‘pearson’).

## 3. Results

### 3.1. Assignment of tagged individuals to functional groups

The first axis of the PCA (figure 3, table 2) contrasted two main categories of behaviour, with foraging being expressed positively and internal behaviours negatively. Along this first axis, the group of ‘foragers’ was clearly discriminated from other workers that were more likely to stay inside the nest. In addition, a significant number of individuals exhibited an intermediate behaviour, spending as much time in the nest as in the foraging area (figure 3). We classified these individuals as being ‘intermittent-foragers’. The second axis opposed grooming behaviours and movement, which were projected positively, with inactivity and brood care, which were projected negatively. This second axis allowed us to distinguish ‘domestics’, which are highly mobile ants engaged in hygienic behaviours, from the other internal workers. The third axis was positively associated with brood care or feeding on prey and negatively associated with inactivity and trophallaxis. The third axis thus allowed to discriminate ‘nurses’ from ‘inactive’ workers. Based on this PCA analysis and the existing literature (see §4), we retained the following five functional groups: foragers, intermittent-foragers, domestics, nurses and inactives. We classified any tagged individual that spent at least 5% of its time caring for the brood as a ‘nurse’ (*n* = 82 nurses in total for the five colonies). For all other tagged workers, we assigned each individual to one of the four functional groups, resulting in 148 foragers, 62 intermittent-foragers, 100 domestics and 99 inactives (see electronic supplementary material, table S1 about the centroid-clustering method for the assignment of individuals).

**Table 2.** Loading of the PCA. Only the components with a s.d. greater than 1 are shown.

behaviour	component 1	component 2	component 3
forage	0.563	0.262	0.141
brood care	−0.288	−0.22	0.656
self-grooming	−0.416	0.388	0
allogrooming	−0.261	0.364	0
trophallaxis	−0.146	0.365	−0.27
feed	−0.283	0	0.555
move	−0.429	0.231	−0.142
inactive	−0.323	−0.642	−0.383
prop. of var.	35.18%	15.77%	12.64%

### 3.2. Necrophoresis

Necrophoresis typically involves three successive steps for which we measured the contribution of each tagged worker. First, the ants contacted the corpse with the tips of their antennae. Then, some workers successively displaced the corpse by grasping it with their mandibles and transporting it inside the nest, closer to the entrance. Finally, one or a few workers discarded the cadaver by moving it from the inside to the outside of the nest.

#### 3.2.1. Contact with corpses

On average, a sporulating cadaver was discarded three times faster than an uninfected corpse (mean discarding time ± s.d.: uninfected cadavers = 49.5 ± 39 min, *n* = 49: sporulating cadavers = 16.4 ± 11.8 min, *n* = 50). As a result, workers had less time to encounter sporulating bodies inside the nest, which resulted in only half of the tagged workers contacting them (49.7%; 244 out of 491 ants). Conversely, the slower removal of uninfected corpses allowed them to be encountered by 78% of tagged workers (381 out of 491 ants).

**Figure 4.**
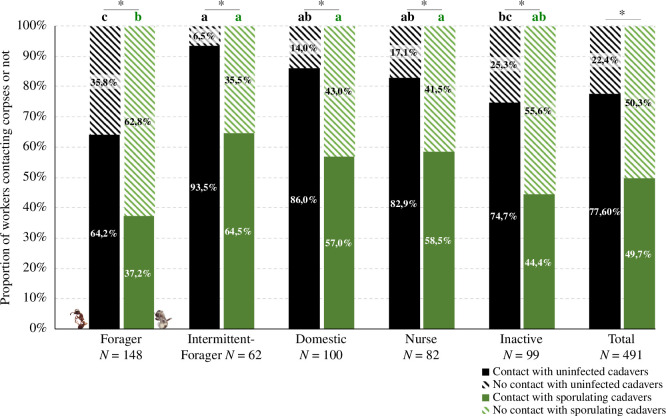
Proportion of tagged workers contacting or not the corpses per functional group. An individual is considered as having contacted the corpse when touching it at least once. Black bars and letters: uninfected corpses. Green bars and letters: sporulating corpses. Full bars: come in contact with cadavers at least once. Striped bars: never contacted cadavers. Significantly different proportions among groups share no common letters (Pearson *χ*^2^-test followed by a pairwise nominal independence test as post hoc). Above each functional group, significantly different proportion within group between the uninfected and sporulating conditions are displayed with a star (McNemar tests).

In each functional group, the proportion of ant individuals contacting a corpse was always significantly higher towards uninfected cadavers (figure 4, McNemar test, d.f. = 1, forager *p* < 0.0001, intermittent-forager *p* < 0.001, domestic *p* < 0.0001, nurse *p* < 0.01, inactive *p* < 0.0001). The functional groups also differed significantly in the proportion of individuals contacting an uninfected corpse (*χ*^2^-test, *χ*² = 30.243, d.f. = 4, *p* < 0.001) or a sporulating cadaver (*χ*² = 20.537, d.f. = 4, *p* < 0.001). In all groups, the majority of workers touched the uninfected cadavers, with intermittent-foragers showing the highest participation rate (93.5%; figure 4). Foragers were less likely to contact uninfected corpses than the other groups (figure 4, post hoc for *χ*^2^-test: forager versus intermittent-forager, *p* < 0.001; forager versus domestic, *p* < 0.01; forager versus nurse, *p* < 0.05), with the exception of inactive ants, which were also fewer to touch these cadavers. More than half of the intermittent-foragers, domestics or nurses touched the sporulating bodies. The group of foragers showed the lowest proportion of ants contacting sporulating corpses (figure 4, post hoc for *χ*^2^-test: forager versus intermittent-forager, *p* < 0.001; forager versus domestic, *p* < 0.05; forager versus nurse, *p* < 0.05; forager versus inactive, n.s.).

In terms of the individual level of exposure to cadavers, tagged workers in each functional group always made more contacts with the uninfected corpses than with the quickly discarded sporulating ones (table 3, Wilcoxon, *W* = 72,216, *p* < 0.0001). In addition, the number of contacts with uninfected or sporulating cadavers differed significantly between the functional groups (table 3, Kruskal–Wallis test, d.f. = 4, uninfected *p* < 0.0001, sporulating *p* < 0.01). Intermittent-foragers made an average number of contacts with uninfected corpses that was significantly greater than those of foragers, nurses and inactive individuals (table 3, paired Nemenyi test; intermittent-forager versus forager, *p* < 0.0001; intermittent-forager versus nurses, *p* < 0.001; intermittent-forager versus inactives, *p* < 0.0001). Although the proportion of foragers contacting uninfected cadavers was relatively small (37% in figure 4), they showed the second-highest contact number (table 3) because some individuals exhibited this behaviour repeatedly. Likewise, when sporulating corpses were introduced, intermittent-foragers made more contacts with corpses than foragers and inactives (table 3, paired Nemenyi test; forager versus intermittent-forager, *p* < 0.01, intermittent-forager versus inactive, *p* < 0.01). Domestic individuals also had a significantly higher number of contacts with sporulating corpses compared with foragers and inactive individuals (table 3, paired Nemenyi test; domestics versus forager, *p* < 0.05; domestics versus inactive, *p* < 0.01).

**Table 3.** Number of contacts with cadavers per worker per functional group. Mean number of contact ± s.d. Significantly different post hoc comparisons among groups share no common letters (Kruskal-Wallis followed by paired Nemenyi test, *α* = 0.05, Tukey correction).

functional group	uninfected cadavers		sporulating cadavers	
	mean ± s.d.	test	mean ± s.d.	test
forager *n* = 148	6.2 ± 9.5	a	1.4 ± 2.7	a
intermittent-forager *n* = 62	9.0 ± 7.4	b	3.4 ± 4.8	b
domestic *n* = 100	5.6 ± 6.3	ab	2.5 ± 3.1	b
nurse *n* = 82	3.8 ± 3.3	a	1.7 ± 2.1	ab
inactive *n* = 99	3.4 ± 5.5	a	1.1 ± 1.8	a
total *n* = 491	5.4 ± 7.3	*χ*² = 17.67 *p* < 0.001	1.9 ± 3.0	*χ*² = 17.57 *p* < 0.001

**Table 4.** Individual probability of displacing an encountered cadaver per functional groups. Mean probability ± s.d. *N* = number of tagged ants having encountered at least one corpse. Significantly different post hoc comparisons among groups share no common letters (Kruskal-Wallis followed by paired Nemenyi test, *α* = 0.05, Tukey correction).

functional group	uninfected cadavers			sporulating cadavers		
	*N*	mean ± s.d.	test	*N*	mean ± s.d.	test
forager	95	0.036 ± 0.088	a	55	0.124 ± 0.263	a
intermittent-forager	58	0.059 ± 0.119	ab	40	0.193 ± 0.245	a
domestic	86	0.012 ± 0.043	b	57	0.159 ± 0.179	a
nurse	68	0.009 ± 0.052	b	48	0.148 ± 0.200	a
inactive	74	0.016 ± 0.052	ab	44	0.114 ± 0.188	a
total	381	0.025 ± 0.075	*H* = 17.23 *p* < 0.01	244	0.146 ± 0.193	*H* = 7.313 *p* = 0.101

#### 3.2.2. Displacement and discarding of corpses

**Figure 5.**
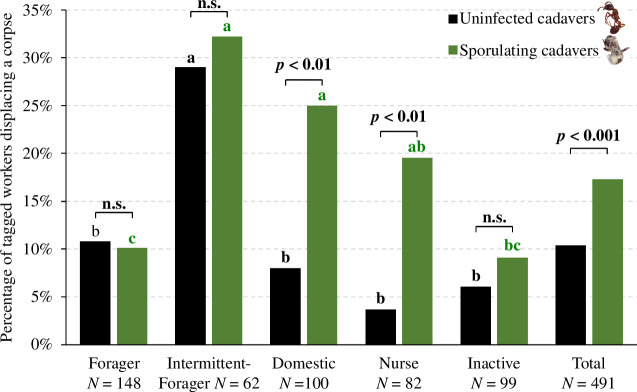
Percentage of tagged workers in each functional group that transported at least one cadaver. Black bars and letters: uninfected cadavers. Green bars and letters: sporulating cadavers. Significantly different post hoc comparisons between groups share no common letters (*χ*^2^-test followed by pairwise nominal independence test as post hoc, *α* = 0.05, *fdr* correction). Above each functional group, lines with the corresponding *p*-value indicate the results of the McNemar test between the sporulating and uninfected condition within each functional group.

While most workers simply walked by the dead nestmate and briefly touched it with their antennae, some individuals grabbed the corpse and transported it, usually to a location closer to the nest exit (number of displacements per cadaver, mean ± s.d.: uninfected corpse = 4.6 ± 3.5, sporulating corpse = 5.0 ± 3.2). These displacements were repeated until one or a few workers discarded the corpse outside the nest. Of the tagged workers who encountered uninfected and sporulating corpses, 13.6% and 35.1%, respectively, responded to the stimulus and displaced the corpses. The probability distribution differed significantly according to the infectiousness of cadavers, with individuals being more eager to move sporulating corpses (Wilcoxon signed-rank test, *W* = 35,191, *p* < 0.0001). A significantly higher percentage (17.3%) of the tagged workers participated in the transport of sporulating bodies compared with uninfected ones (10.4% of tagged ants) (figure 5, McNemar test; *χ*² = 11.344, d.f. = 1, *p* < 0.001). The percentage of intermittent-foragers (29%) displacing at least one uninfected corpse was significantly higher than for any other functional group (figure 5, post hoc test for *χ*^2^-test; intermittent-forager versus forager, *p* < 0.01; intermittent-forager versus domestics, *p* < 0.01; intermittent-forager versus nurse, *p* < 0.001; intermittent-forager versus inactive, *p* < 0.001). Likewise, in the case of sporulating cadavers, the percentage of intermittent-foragers taking part in their displacement was significantly greater than that of foragers or inactives (figure 5, post hoc for *χ*^2^-test; intermittent-forager versus forager, *p* < 0.001; intermittent-forager versus inactive, *p* < 0.05). However, the proportion of intermittent-foragers displacing sporulating corpses did not differ from that of domestic and nurses (intermittent-forager versus domestics, *p* = 0.514; intermittent-forager versus nurse, *p* = 0.171). This was due to a higher percentage of domestic workers and nurses moving sporulating corpses compared with uninfected ones (figure 5, McNemar test; domestic: *χ*² = 9.482, d.f. = 1, *p* < 0.01; nurse: *χ*² = 7.579, d.f. = 1, *p* < 0.01).

Finally, the likelihood that a worker moves a cadaver upon encounter was strongly influenced by its infectiousness (table 4). For uninfected corpses, these probabilities were consistently low but nevertheless varied significantly between functional groups (table 4, Kruskal-Wallis test, *χ*² = 21.422, d.f. = 4, *p* < 0.01, ties method = average). Individuals belonging to the forager and intermittent-forager groups were the most responsive to corpse-related stimuli, as they were most likely to displace them upon encounter (table 4, post hoc Nemenyi test, forager versus domestic: *p* < 0.01, forager versus nurse: *p* < 0.05, forager versus inactive: *p* = 0.51). However, the higher probability of intermittent-foragers did not statistically differ from those of the other groups, probably due to their small sample size and high interindividual variance. In the case of the sporulating cadavers, the probabilities of moving these infectious items were consistently high but did not differ between functional groups (table 4, Kruskal-Wallis test, *χ*² = 7.313, d.f. = 4, *p* = 0.101, ties method = average).

#### 3.2.3. Individual specialization

**Figure 6.**
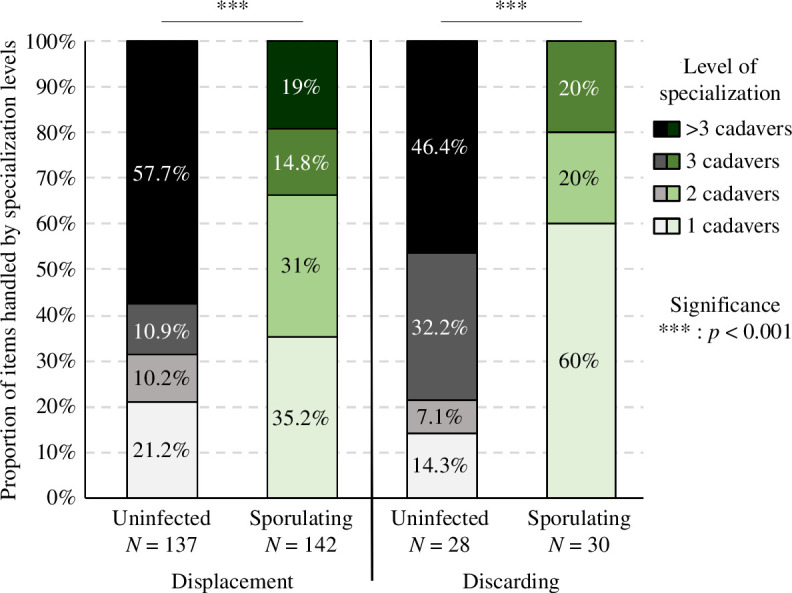
Contribution of individuals to necrophoresis according to the level of specialization. Shades of black and white: uninfected corpses. Shades of green: sporulating corpses. Significantly different proportion between conditions are displayed with stars (*χ*^2^-tests). See electronic supplementary material, table S3 for details about individual specialization across functional groups.

As for the individual specialization in necrophoresis, 22 out of the 51 tagged workers having displaced uninfected corpses made several transports and contributed to 78.8% of the displacements (figure 6). Specialization followed a similar pattern for sporulating corpses, as 35 out of the 85 tagged ants that moved corpses made more than one displacement and were responsible for 64.8% of these transports (figure 6). Thus, the relative contribution of these specialized workers to corpse displacements was higher for uninfected cadavers than for sporulating ones (figure 6, *χ*^2^-test, *χ*² = 47.5, d.f. = 3, *p* < 0.0001). Regarding the discarding of corpses, 6 out of 11 tagged ants removed several uninfected corpses, whereas only 5 out of 23 tagged ants rejected several sporulating cadavers. As a result, 85.7% of uninfected corpses were discarded by individuals engaged several times in necrophoresis, whereas only 40% of sporulating corpses were discarded by workers removing more than one cadaver. Thus, the proportion of cadavers discarded by specialized workers was significantly greater for uninfected cadavers compared with sporulating cadavers (figure 6, *χ*^2^-test, *χ*² = 24.5, d.f. = 3, *p* < 0.0001). Looking at the functional groups, in the case of uninfected cadavers, these short-term specialists were mainly foragers and intermittent-foragers, representing 15 out of the 22 individuals and 6 out of the 7 individuals who, respectively, moved and discarded several uninfected corpses (electronic supplementary material, table S3). In contrast, short-term specialization in the necrophoresis of sporulating cadavers occurred mainly among domestic and intermittent-foragers, which represented 21 out of 35 individuals and 4 out of 5 individuals that, respectively, moved and discarded more than one sporulating corpse.

### 3.3. Mortality

**Figure 7.**
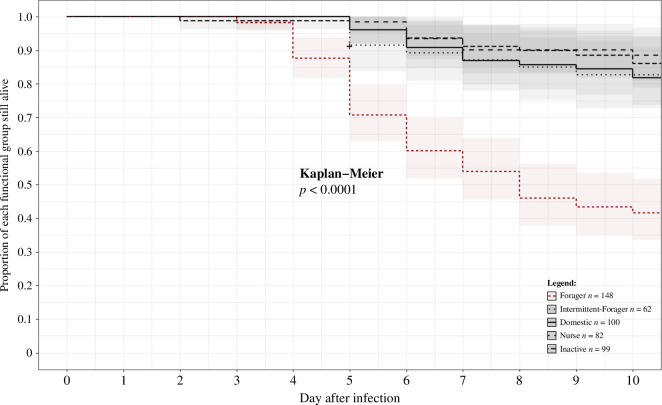
Survival curve per functional group. Mortality was calculated over the individuals that still bore their tag at the end of experiment. X-scale represents days after introduction of contaminated carcasses. The Kaplan–Meier test shows a significant difference in the mortality dynamics of foragers (in red) compared with other functional groups (in black).

We found no significant difference in the survival curves of tagged workers across the five colonies (electronic supplementary material, figure S5, Kaplan-Meier test, n.s.). Colony mortality at the end of the experiment ranged from 23.5% to 38.3% of the total worker population, with an average of 30.5% (± 5%; *n* = 5 colonies). Before the introduction of sporulating cadavers (days 5–11), the daily rate of colonial mortality was low (mean over the 7 days = 0.6% of the colony ± 0.11, *n* = 5), with no signs of sporulation over corpses. Colony exposure to sporulating bodies increased daily mortalities, peaking at 9.47% (± 1.9%) on day 16. Most of the deceased ants (86%) died on days 16–21 after the introduction of infectious cadavers (electronic supplementary material, figure S6). By checking the cause of death, we found that the majority of those cadavers (90.3%; 139 out of 154 corpses sampled out of three colonies) sporulated into the ‘white muscadine’ typical of *B. bassiana* infection. Mortalities differed between functional groups, with foragers showing the lowest survival curves (figure 7, Kaplan–Meier test, *p* < 0.0001). The mortality rates of all other functional groups were similar (figure 7). Finally, we compared the proportion of tagged workers that died depending on their previous level of exposure to corpses, i.e. no contact with corpses, contacting cadavers between 1 and 60 s, and for more than 60 s. We found no significant difference in mortality of workers according to their duration of exposure to non-infected corpses (*n* = 377, *χ*² test, n.s.). On the contrary, the proportion of workers that died after handling sporulating cadavers for more than 60 s was significantly higher than for workers that never contacted these cadavers (*n* = 377, *χ*² test, *p* < 0.05; post hoc: 0 seconds versus more than 60 s, *p* < 0.05).

### 3.4. Prophylactic self-grooming and mobility

**Figure 8.**
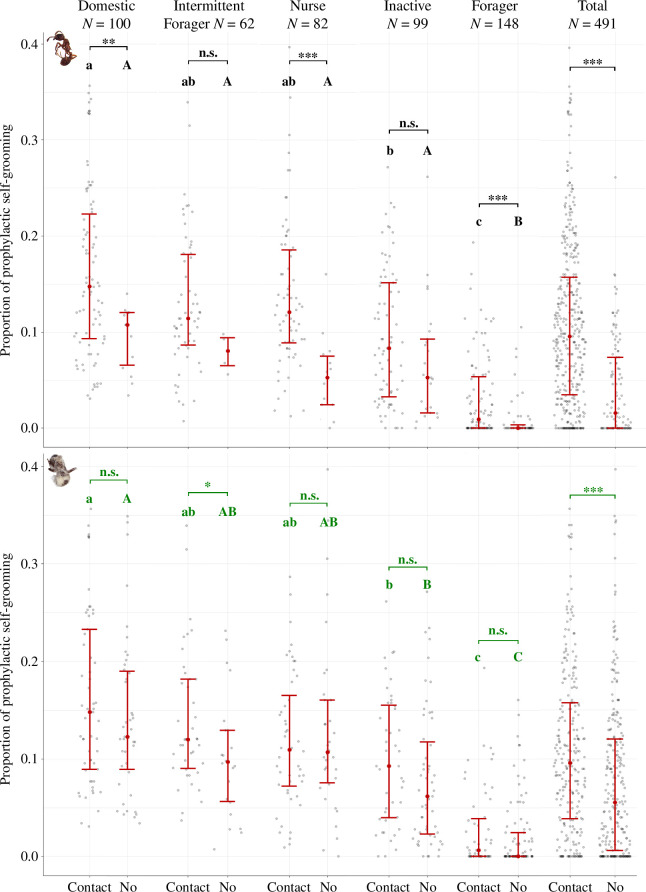
Proportion of prophylactic self-grooming per functional group depending on whether individuals later had contact with cadavers. (*a*) Uninfected condition. (*b*) Sporulating condition. The proportion of time spent performing self-grooming was measured for each tagged ant during behavioural analyses, prior to the introduction of corpses. Each functional group is divided into individuals who touched the cadavers at least once (Contact) and workers who never touched them (No). Significant differences within group are indicated by stars (Wilcoxon test, *α* = 0.05). Significantly different post hoc comparisons among functional groups share no common letters (black = uninfected condition, dark green = sporulating condition, lowercase letters = contact, capital letters = no contact. Kruskal–Wallis test followed by paired Nemenyi as post hoc, *α* = 0.05, Tukey correction).

Self-grooming performed by workers prior to exposure to pathogens may be considered a strategy of individual prophylaxis. Interestingly, workers who had contact with corpses previously showed more prophylactic grooming than those who had never contacted corpses (figure 8, Wilcoxon test, *W* = 10,7, *p* < 0.0001 for uninfected corpses, *W* = 22,9, *p* < 0.001 for sporulating corpses). We examined whether this difference in prophylactic grooming persisted when considering each functional group separately. Forager, domestic and nurse individuals who further contacted uninfected corpses were showing, prior to the introduction of cadavers, more frequent prophylactic grooming compared with the individuals who never contacted corpses (figure 8, Wilcoxon test: forager, *W* = 1671, *p* < 0.001; domestic, *W* = 311, *p* < 0.01; nurse, *W* = 135, *p* < 0.0001). Inactive and intermittent-foragers showed similar trends of prophylactic grooming, but differences were only marginally significant (Wilcoxon test: *W* = 687, *p* = 0.055 for inactive; *W* = 56, *p* = 0.085 for intermittent-foragers). A similar picture was obtained for sporulating corpses since the individuals that contacted infectious corpses also exhibited more prophylactic self-grooming. This trend was, however, not significant when considering each functional group separately (Wilcoxon test: forager, *W* = 2228, *p* = 0.16; domestic, *W* = 1063, *p* = 0.26; nurse, *W* = 836, *p* = 0.86; inactive, *W* = 970, *p* = 0.09), excepting for the intermittent-foragers group (Wilcoxon test, *W* = 306, *p* = 0.049). Interestingly, self-grooming was positively correlated with movement in the behavioural profiles of tagged workers (electronic supplementary material, figure S7, Pearson’s correlation, *n* = 491, *T* = 14.9, *R* = 0.56, *p* < 0.001). Workers who had contact with corpses were also more mobile than those who never touched them (Wilcoxon test, uninfected corpses: *W* = 13,6, *p* < 0.0001; sporulating corpses: *W* = 24, *p* < 0.0001). We investigated whether this difference persisted at the level of functional groups, in an analogous way to prophylactic self-grooming. Foragers were the only group in which individuals who came into contact with uninfected corpses were significantly more mobile than those who did not (Wilcoxon test: forager, *W* = 1572, *p* < 0.001; intermittent-forager, *W* = 144, *p* = 0.44; inactive, *W* = 950, *p* = 0.85; domestic, *W* = 502, *p* = 0.32; nurse, *W* = 341, *p* = 0.097). In the case of sporulating corpses, only the domestic group showed a difference in mobility between workers who later touched these corpses and those who never touched them (Wilcoxon test: domestic, *W* = 933, *p* = 0.042; forager, *W* = 2355, *p* = 0.40; intermittent-forager, *W* = 401, *p* = 0.57; inactive, *W* = 1151, *p* = 0.68; nurse, *W* = 890, *p* = 0.49).

## 4. Discussion

According to our results, the work organization in *M. rubra* colonies is based on five functional groups: foragers, intermittent-foragers, domestics, nurses and inactive ants. As with many ant species [61], foragers spend most of their time exploiting resources, exploring or defending their nest surroundings. Interestingly, we identified a group of intermittent-foragers, which divide their time between the nest and the foraging area. A similar group has been identified in the species *Neoponera apicalis* [57,58]. *Myrmica rubra* intermittent-foragers could also be similar to the group of nest maintenance and midden workers (e.g. in *Pogonomyrmex* harvester ants [12]), which also split their time between in-nest and out-of-nest activities. Indeed, nest maintenance workers modify nest chambers and reject soil particles, whereas midden workers discard waste items on rubbish piles outside the nest. As for the domestics, they are highly active and mobile workers that frequently engage in social and self-grooming behaviours and that show a behavioural profile similar to the workers described in other ant species as ‘patrollers/groomers’ [51], ‘domestics’ [57,58] or ‘cleaners’ [50]. The fourth group of nurses takes care of larvae and frequently shows feeding behaviour [60]. Finally, as documented in many ant species [52,62], the group of inactive workers remains motionless most of the time inside the nest, sometimes considered as a reserve caste that is activated when workload increases [63].

These functional groups are engaged to different extents in the necrophoresis process. Necrophoresis typically consists of three successive steps: workers first contact the corpse with their antennae, then some workers grasp it with their mandibles and transport it closer to the nest entrance, and finally, a worker discards the cadaver outside the nest [15–17]. Remarkably, workers were more likely to contact corpses than displace or discard them. By limiting their interactions with cadavers to contacts with the tip of antennae, workers make it possible to assess the presence of waste items and their pathogenicity while avoiding the huge risks of getting infected by transporting potentially infectious items. Overall, our results clearly demonstrate that the disposal of nestmate cadavers relies on some functional groups which are more likely to detect, move and discard potentially hazardous items. The functional groups that contributed the most and least to the discarding of corpses were the intermittent-foragers and inactive workers, respectively. Unexpectedly, fungal contamination of corpses induced a wider commitment of more functional groups, as the achievement of necrophoresis was extended to other functional groups such as domestic workers or even nurses. The observed work organization supports the concept of spatial modules [22] because the functional group most involved in the necrophoresis process shares the same spatial location as the task to be achieved. Indeed, intermittent-foragers appear to be the best ergonomically suited to discard corpses, as they occupy an intermediate location between the inside and outside of the nest. Our results are also in line with previous studies showing the commitment of external workers to necrophoresis [16,64]. Indeed, *M. rubra* foragers moved and discarded uninfected corpses, although their contribution was lower than that of intermittent-foragers because of their shorter stay in the nest and thus reduced opportunities to encounter cadavers. Concerning the domestics, these highly mobile workers, who frequently contacted corpses, responded weakly to corpse-related stimuli and thus contributed little to the discarding of uninfected bodies. However, when corpses posed a major hygienic threat to the colony, domestics were much more responsive to sporulating bodies and became highly engaged in discarding them. This finding confirms that task allocation is a flexible adaptive trait, which is shaped not only by the spatial location of workers but also by changes in their individual probability of responding to critical stimuli. The commitment of a wider range of functional groups to discarding of sporulating corpses suggests that ants have a lower response threshold to stimuli associated with fungus-infected cadavers than to non-infectious corpses. Even nurses made a significant number of contacts with sporulating cadavers, and some even engaged in their displacement. The contribution of nurses to the displacement of infectious corpses was quite unexpected given their critical role in rearing disease-susceptible larvae. This raises the question of how the pathogenicity of waste could force a worker out of its spatial zone for the sake of a rapid nest sanitization. Inactive ants consistently exhibited the lowest number of contacts and a notably low probability of moving corpses, as expected from their typically weak responsiveness to various stimuli [52,65]. This finding indicates that undertakers are not ‘reserve’ individuals that remain inactive most of the time and that maintain high thresholds for stimuli not associated with the management of deceased nestmates.

Ant societies adapt hygienic responses to waste pathogenicity in a context-dependent manner. Outside the nest, foragers systematically avoid touching and retrieving hazardous prey contaminated with entomopathogenic fungi [32,40,66]. Inside the nest, workers are more likely to come into contact and move prey or nestmate corpses that are covered with spores or have died from infection to quickly discard them from the nest [30,32,67]. This study confirms that the active handling and discarding of contaminated waste by a large number of workers belonging to multiple functional groups overrides avoidance strategies as a means of resisting pathogens. Nestmate corpses killed by *Beauveria* fungus were discarded more quickly than uninfected cadavers, thereby lowering the level of colony exposure to the pathogen. Furthermore, our findings indicated that workers that engaged in prolonged contacts with sporulating corpses exhibited a significantly higher mortality rate than ants having not encountered these infectious corpses. This raises the question of how colonies have evolved a trade-off between limiting the number of ants specialized in necrophoresis at high individual risks of getting infected and expanding this hazardous activity to a large part of the workers’ population in order to speed up the process of waste discarding and nest sanitization. We found that this trade-off is influenced by the pathogenicity of cadavers, with specialization in corpse removal among individuals or functional groups being less likely to emerge in the case of sporulating corpses. When corpses were not infectious, as reported by Jullian and Cahan [15] in leaf-cutting ants, some *M. rubra* individuals were involved in several successive necrophoric events and were responsible for the majority of corpse displacement and discarding. On the other hand, due to the general increase in individual probabilities to deal with sporulating cadavers, the contribution of specialist individuals to necrophoresis was lowered. Individual specialization is enabled when an individual remains at a given task until the required behaviour is successfully performed and its response threshold to the task-associated stimuli is lowered [10]. This implies that appropriate stimuli should remain present as long as self-reinforcing learning processes and individual specialization are in progress [68]. Our findings show that the emergence of individual specialists indeed depends on the intensity of corpse-related stimuli and, in particular, on how it changes over time. We found that a faster discarding of sporulating corpses accelerates the decrease in stimulus intensity, thus reducing the opportunities for workers to encounter multiple corpses and ultimately preventing ant individuals from becoming progressively specialized in necrophoresis. Individual specialization is often considered critical for the ergonomic efficiency of insect societies because it accelerates task completion. Our results suggest that the reverse is also true: the dynamics of task completion can determine the level of individual specialization by limiting the time during which individual specialization can emerge. In the case of necrophoresis, the balance seems to be tilted towards more individual specialization in the management of uninfected cadavers and towards faster removal by a wider population of workers for sporulating items.

The management of wastes as potential sources of pathogens is known to incur mortality risks to the workers involved [30,40,69]. We expected the mortality rates to increase with the level of involvement of functional groups in corpse displacement and discarding. Quite unexpectedly, given their relatively limited participation in necrophoresis, foragers experienced far more casualties than any other functional group, with high sporulation rates that left no doubt as to the cause of death. Five non-mutually exclusive hypotheses can explain this result. (i) An underestimated exposure of foragers to pathogens since, once discarded, cadavers remain for some time in the foraging area and can be further contacted by foragers. (ii) Fewer social contacts and allogrooming received by foragers due to their location outside the nest, thereby giving more time to *Beauveria* spores to penetrate the cuticular barrier and invade the ant body [39,70,71]. (iii) An immunosenescence of foragers suffering from an age-related decline in immunocompetence (as shown in honeybees: [72,73]; bumblebee: [74]). (iv) A decline in worker corpulence with foraging activity [59,75], resulting in lower energy reserves available to foragers to fight infection. (v) The differences in olfactory perception between foragers and internal workers [76] influencing their response to sporulating corpses stimuli. Interestingly, all the other functional groups (intermittent-foragers, nurses and domestics) involved in the necrophoresis of sporulating bodies experienced limited lethality, never exceeding 20% of their population after 21 days of experiment. Contact-transmitted pathogens, such as *Beauveria*, require a certain threshold of conidia *per capita* to kill their hosts [77]. We assume that the spread of a certain number of conidia over a large population of workers across multiple functional groups engaged in corpse removal may have diluted the pathogen load *per capita* below the lethal threshold. Over time, exposure to sub-lethal doses of fungal conidia could even induce immune priming in workers, thereby strengthening their resistance to subsequent pathogen exposure [78] but see [79]. However, a wider commitment of the workers’ population in the removal of sporulating wastes has drawbacks: it increases the likelihood that key individuals, such as queens and larvae, will come into contact with nestmates who have previously touched a sporulating item and facilitates pathogen dissemination throughout the nest by a high number of spore-bearing ant vectors.

The present study contributes to improving our understanding of how the level of worker specialization and the dynamics of waste discarding may be related to waste pathogenicity. One may wonder whether, when considering a gradient of pathogenicity, the trend towards a generalized involvement of workers in necrophoresis is maintained, enhanced or replaced by alternative strategies. In particular, ant societies can be exposed to pathogens that are highly infectious at low spore amounts *per capita* [80]. If the trend observed in this study is enhanced, any encounter with such highly infectious items should be associated with a very low response threshold, thereby triggering immediate handling by a worker and a steep decline in pathogen load inside the nest. However, the benefits of faster corpse discarding due to the engagement of many workers may be outweighed by the high mortality rate of the involved individuals. We can speculate that an alternative strategy for dealing with waste contaminated by highly infectious pathogens would be for most ant workers to systematically avoid contacting these items and for a very limited number of individuals to handle them, even at the expenses of their slower removal from the nest. Relying on a few specialists to deal with these highly infectious items could be the best strategy for limiting colony-level mortality costs. However, it would be effective only on the conditions that contaminated items are confined to a limited nest area and that colony organizational immunity restrains social interactions with these specialists. The ant host that is engaged in an evolutionary arms race against highly infectious pathogens may benefit from the existence of some workers—ideally, foragers with shorter life expectancies—who sacrifice themselves for the sake of the whole colony. According to this last scenario and as shown here for the necrophoresis of non-infectious corpses, higher levels of specialization could thus be expected at both ends of the spectrum of waste pathogenicity.

Hygienic behaviours are induced by exposure to pathogens; however, prophylactic behaviour can also be performed in the absence of direct health threats, as shown in ants [27,32,81], bees [82,83] and termites [84]. In addition to waste management, grooming is the first effective line of defence against contact-transmitted pathogens [38,39,71]. Interestingly, when comparing workers who touched cadavers with those who did not, the former showed higher baseline levels of self-grooming prior to the insertion of corpses into the nest. These ‘hygienist’ individuals were also highly mobile inside the nest and therefore were probably more likely to detect and contact wastes. When considering the group of domestics, nurses or foragers separately, prophylactic grooming behaviour was more common in ants who later touched uninfected corpses. A significantly higher occurrence of prophylactic grooming was also observed in the intermittent-foragers who later contacted sporulating cadavers. In insect societies, individuals from different lineages can differ in their overall task performance [85]. Because of the high level of polygyny in *M. rubra* ant species [42,86], variation in the genetic background of ant workers may account for such differences in the performance of hygienic behaviour. As in bees [87], individuals responsible for grooming, waste removal and necrophoresis could share a common ‘hygienic’ lineage. Further studies should investigate whether these hygienist individuals are also characterized by a higher ability to detect any foreign objects over the cuticle or inside the nest, explaining their higher self-grooming performance prior to any infection. The presence of hygienist individuals appears to be another aspect of social immunity in ants that deserves further investigation.

In conclusion, our study has underlined the trade-off between a reduction of the number of individuals exposed to pathogens, which arises from the emergence of short-term specialists, and an increased efficiency at the colony level, which arises from a larger population of nestmates working together to speed up nest sanitization. Investigating how waste pathogenicity can influence the level of individual specialization and collective dynamics of waste disposal can provide a deeper understanding of the evolutionary interplay between pathogen-driven selective forces and work organization in insect societies.

## Data Availability

Data and metadata are available through the dryad repository [88]. Supplementary material is available online [89].
